# Active and passive sexual roles that arise in *Drosophila* male-male courtship are modulated by dopamine levels in PPL2ab neurons

**DOI:** 10.1038/srep44595

**Published:** 2017-03-15

**Authors:** Shiu-Ling Chen, Yu-Hui Chen, Chuan-Chan Wang, Yhu-Wei Yu, Yu-Chen Tsai, Hsiao-Wen Hsu, Chia-Lin Wu, Pei-Yu Wang, Lien-Cheng Chen, Tsuo-Hung Lan, Tsai-Feng Fu

**Affiliations:** 1 Department of Applied Chemistry, National Chi Nan University, 54561 Nantou, Taiwan; 2 Department of Life Science, Fu Jen Catholic University, 24205 New Taipei City, Taiwan; 3 Department of Life Science and Life Science Center, Tunghai University, 40704 Taichung, Taiwan; 4 Department of Biochemistry and Graduate Institute of Biomedical Sciences, College of Medicine, Chang Gung University, 33302 Taoyuan, Taiwan; 5 Department of Neurology, Linkou Chang Gung Memorial Hospital, 33305 Taoyuan, Taiwan; 6 Graduate Institute of Brain and Mind Sciences, College of Medicine, National Taiwan University, 10051 Taipei, Taiwan; 7 Department of Medical Laboratory Science and Biotechnology, Chung Hwa University of Medical Technology, 71703 Tainan, Taiwan; 8 School of Medical Laboratory Science and Biotechnology, College of Medical Science and Technology, Taipei Medical University, 11031 Taipei, Taiwan; 9 Department of Psychiatry, School of Medicine, National Yang Ming University, 11221 Taipei, Taiwan; 10 Department of Psychiatry, Taichung Veterans General Hospital, 40705 Taichung, Taiwan

## Abstract

The neurology of male sexuality has been poorly studied owing to difficulties in studying brain circuitry in humans. Dopamine (DA) is essential for both physiological and behavioural responses, including the regulation of sexuality. Previous studies have revealed that alterations in DA synthesis in dopaminergic neurons can induce male-male courtship behaviour, while increasing DA levels in the protocerebral posteriolateral dopaminergic cluster neuron 2ab (PPL2ab) may enhance the intensity of male courtship sustainment in *Drosophila*. Here we report that changes in the ability of the PPL2ab in the central nervous system (CNS) to produce DA strongly impact male-male courtship in *D. melanogaster*. Intriguingly, the DA-synthesizing abilities of these neurons appear to affect both the courting activities displayed by male flies and the sex appeal of male flies for other male flies. Moreover, the observed male-male courtship is triggered primarily by target motion, yet chemical cues can replace visual input under dark conditions. This is interesting evidence that courtship responses in male individuals are controlled by PPL2ab neurons in the CNS. Our study provides insight for subsequent studies focusing on sexual circuit modulation by PPL2ab neurons.

Courtship is an instinctive behaviour whereby animals utilize their sensory perception to recognize a conspecific and suitable partner. It has been determined that dopamine (DA) modulates the intensity of male courtship in *Drosophila*^1^,^2^, and that DA is associated with the regulation of female sexual receptivity^3^. Moreover, an increase in DA levels in a subset of PPL2ab neurons is sufficient to enhance courtship sustainment in both young and aged males^2^. On the other hand, down-regulation of DA levels in PPL2ab neurons significantly decreases the sustainment of courtship^2^. A previous study has also shown that increasing DA levels in most dopaminergic (DAergic) cells may induce intense inter-male courtship in *Drosophila*^4^. Interestingly, a decrease in DA levels is observed when males experience enhanced attractiveness toward other males during inter-male courtship^5^. In addition, DAergic neurons have been associated with alcohol-induced male-male courtship behaviour^6^. Together, these findings indicate that DA plays an essential role in regulating *Drosophila* sex responses, including those involved in male-male homosexual behaviour.

Sensory cues play key roles in initiating and maintaining courtship behaviour in *Drosophila*^7^,^8^. Previous studies have shown that male courtship performance may be weakened under conditions wherein visual cues are absent^7^,^9^,^10^. Male flies vibrate their wings to generate the so-called “courtship song”, which consists of two different acoustic modes, a pulse song and a sine song. The inter-pulse intervals are important for species specificity, and females reliably distinguish suitable conspecific suitors via these specific acoustic cues^11^. Similarly, sounds produced by the female allow males to detect her presence and further facilitate courtship^12^. Apart from visual and acoustic cues, chemical cues are the most important factors in controlling *Drosophila* courtship behaviour, as flies use chemosensation to make courtship decisions^7^,^8^,^13^,^14^. Mature flies can generate different cuticular hydrocarbons (CHs), which are chemical pheromone cues with different compositions in each gender.

Courtship presentation in *Drosophila* is gender-specific. Specifically, males generally play the active courter role, while females play the passive courtee role. The actions exhibited by the two sexes are thus completely different^15^. In a previous study, we showed that PPL2ab neurons enhance male courtship intensity toward females by increasing the levels of DA^2^. Here we report that changes in DA levels in PPL2ab neurons also strongly impact male-male courtship in *D. melanogaster*. Intriguingly, the dopamine-synthesizing abilities of these neurons appear to affect both the courting activities displayed by males and the sex appeal of males for other males. We observed inter-male courtship behaviour in males with high DA levels in PPL2ab neurons during visually dependent guidance, while preference toward females was unaffected. Interestingly, males with low DA levels in PPL2ab neurons passively embodied the courtee role by releasing the corresponding CHs, thereby enhancing their attractiveness to males with high DA levels in PPL2ab neurons. These findings provide compelling evidence that the brain may regulate active and passive male homosexual responses by controlling DA levels in PPL2ab neurons.

## Results

### Increased DA levels in PPL2ab neurons leads to inter-male courtship behaviour

**Figure 1:**
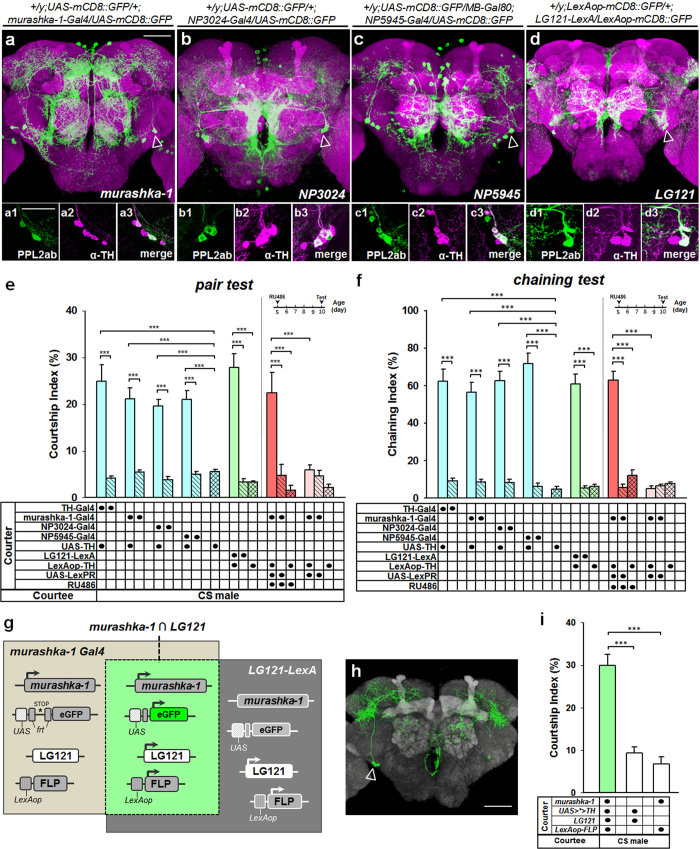
Increased DA in PPL2ab neurons prompts male-male courtship behaviour. Representative images showing the expression patterns of the different driver lines. (**a**) *murashka-1*, (**b**) *NP3024*, (**c**) *NP5945*. and (**d**) *LG121* expression patterns reported by *UAS-mCD8::GFP* in the adult brain (10-day-old) are shown (green in **a–d** shows PPL2ab cell bodies indicated by the arrowheads). The neuropil is immunostained by anti-Discs Large antibody (magenta). PPL2ab neurons targeted by the four independent drivers are dopaminergic, as indicated by TH immunostaining. The scale bars are 20 μm. (**e**,**f**) Ten-day-old mature male flies overexpressing TH in PPL2ab neurons had more frequent male-male courtship responses. There were significant differences in the courtship index (**e**) and chaining index (**f**) in 10-day-old mature males carrying *TH-Gal4* > *UAS-TH, murashka-1-Gal4* > *UAS-TH, NP3024-Gal4* > *UAS-TH, NP5945-Gal4* > *UAS-TH*, or *LG121-LexA* > *LexAop-TH*, and in 5-day-old males carrying *UAS-LexPR;murashka-1-Gal4* > *LexAop-TH* fed 1.5 mM RU486 for 5 days when compared to their corresponding driver- and effector-heterozygous controls. Each column represents the mean of 18 courtship pair tests and 12 chaining tests. (**g**) Strategies for LexA-induced FLP recombinase (FLP) and GAL4 intersectional methods restrict the expression of the driver to PPL2ab neurons. The two gray boxes represent the extents of the *murashka-1* (GAL4) or *LG121* (LexA) expression patterns. (**h**) Micrograph of the intersectional expression patterns of the *murashka-1* and *LG121* drivers. *eGFP* expression is limited to regions wherein both FLP and GAL4 are expressed. The *UAS* > *eGFP* reporter is only expressed in a subset of PPL2ab neurons that co-express both GAL4 and FLP. (**i**) This genetic intersectional approach was used to restrict TH over-expression to PPL2ab neurons and analyze male-to-male courtship intensity. There were significant differences in the courtship indices of *LexAop-FLP* *+* , *murashka-1* X *UAS* > * > *TH*, and *LG121* flies compared to those of the corresponding parental heterozygous flies. Each column represents the mean of 16 tests. Error bars indicate SEM, ^***^P *<* 0.001. Analyses were performed using a one-way ANOVA followed by Tukey’s test or a two-way ANOVA followed by a Bonferroni multiple-comparisons test.

Tyrosine hydroxylase (TH) is an important enzyme in the DA biosynthetic pathway. Overexpression of TH leads to an increase in DA levels^2^. Increasing DA levels in *TH-Gal4-*expressing cells can promote inter-male courtship behaviour^4^, while increasing DA levels in PPL2ab neurons can enhance the intensity of male courtship toward females^2^. To study PPL2ab neurons genetically, we first confirmed that DAergic PPL2ab neurons can be targeted using different PPL2ab expression drivers (*murashka-1-Gal4, NP5945-Gal4, NP3024-Gal4*, and *LG121-LexA*) by performing TH immunostaining (Fig. 1a–d)^2^. We then selectively overexpressed TH in PPL2ab neurons by placing the *UAS-TH* transgene under the control of different PPL2ab-specific expression drivers (*murashka-1-Gal4* > *UAS-TH, NP5945-Gal4* > *UAS-TH, NP3024-Gal4* > *UAS-TH*, and *LG121-LexA* > *LexAop-TH*). In these flies, the courtship index (CI) (Fig. 1e, Supplementary Movie S1) and chaining index (ChI) (Fig. 1f, Supplementary Movie S2) of the progeny was significantly higher than that of the corresponding heterozygous controls.

We also found that increasing DA levels using *TH-Gal4* either ubiquitously or specifically in PPL2ab cells produced similar inter-male CI and ChI values (Fig. 1e and f). This implies that although increases in DA levels in almost all DA cells (*TH-Gal4* > *UAS-TH*) significantly induces inter-male courtship behaviour^4^, PPL2ab neurons might play a more important role that other neurons in this effect. To further confirm the importance of TH in PPL2ab neurons, we induced TH production to yield more DA within PPL2ab neurons in 10-day-old male flies using the drug-inducible *LexPR*/*LexAop* gene expression technique^16^. This approach involves feeding drug-sensitive engineered flies with RU486 to activate overexpression of the TH transgene (*UAS-LexPR*/+; *murashka-1-Gal4*/*LexAop-TH*). Using this approach, we found that the CI and ChI values were also significantly increased in drug-treated males compared to untreated controls during a specific period in adulthood (Fig. 1e and f).

**Figure 2:**
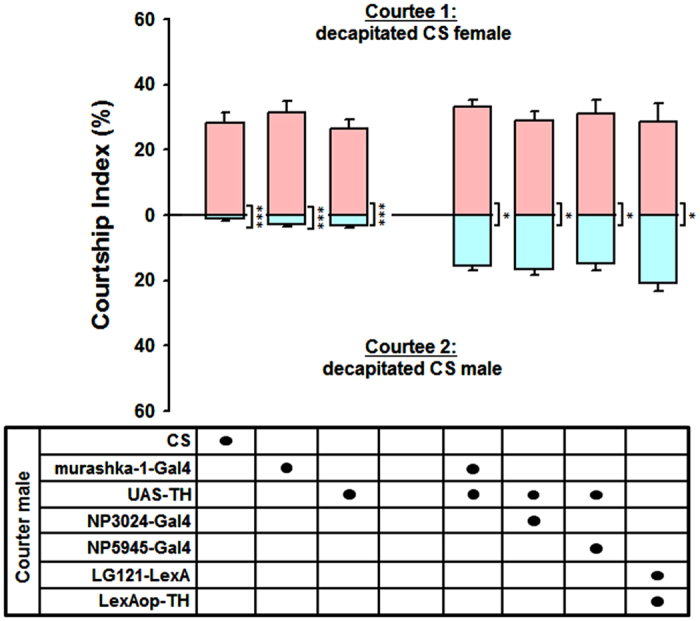
Increased DA in PPL2ab neurons does not alter sexual preference in males. Courtship competition tests indicated that the courtship index in courter males carrying *murashka-1-Gal4* > *UAS-TH, NP3024-Gal4* > *UAS-TH, NP5945-Gal4* > *UAS-TH*, or *LG121-LexA* > *LexAop-TH* was significantly higher for females than for males. Each column represents the mean of 18 competitive courtship assay tests. Error bars indicate + SEM, ^***^P *<* 0.001, ^*^P *<* 0.05. Analyses were carried out using a one-way ANOVA followed by Tukey’s test.

Because the expression patterns of the four PPL2ab drivers used in these experiments were very broad (Fig. 1a–d), we used intersectional genetic strategies to precisely manipulate DA levels specifically in PPL2ab neurons. LexA-induced *FLP*/*frt* recombination was used to optimize TH-targeted expression in an intersectional region by targeting overlapping GAL4 and FLPase activities. FLPase driven by LexA was used to remove *frt-stop-frt* (>*>) from a *UAS-frt-stop-frt-mCD8::GFP (UAS* >* > *GFP*) line in *LG121-*expressing neurons (Fig. 1g). *UAS-mCD8::GFP* was activated by GAL4 in PPL2ab neurons at the intersection of the *LG121* and *murashka-1 (LG121* ∩ *murashka-1*) expression regions (Fig. 1h). This enabled us to use this line of flies for precise behavioural studies in the context of DA changes in PPL2ab neurons. Upon targeting the specific expression of TH to PPL2ab neurons using this approach, there was an obvious induction of sexual responses toward wild type (CS) male targets, as the inter-male CI value was significantly higher than that of the corresponding heterozygous controls (Fig. 1i). Courtship competition tests indicated that the courtship preference of males with high DA levels in PPL2ab neurons toward wild type (CS) female targets was significantly higher than that for CS male targets. In addition, we observed significantly higher male-male CIs when compared to CS, *murashka-1-Gal4*/+, and *UAS-TH*/+ males (Fig. 2).

### Motion cues initiate PPL2ab neuron-mediated male-male courtship behaviour

**Figure 3:**
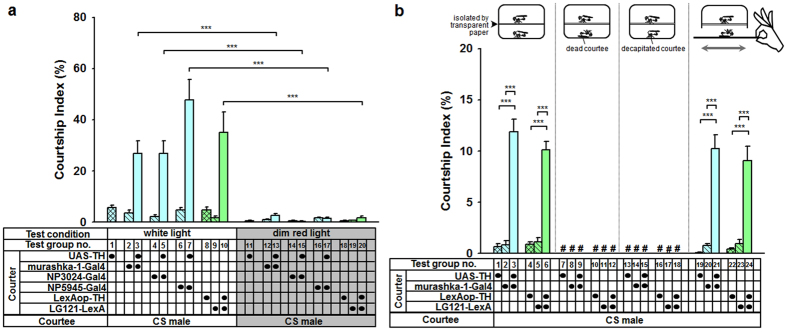
Visual motion cues initiate PPL2ab neuron-mediated male-male courtship behaviour. (**a**) Ten-day-old mature male flies overexpressing TH in PPL2ab neurons displayed visual signal-dependent male-male courtship interactions. There were significant differences in the courtship indices of flies carrying *murashka-1-Gal4* > *UAS-TH, NP3024-Gal4* > *UAS-TH, NP5945-Gal4* > *UAS-TH*, or *LG121-LexA* > *LexAop-TH* compared to their corresponding driver- and effector-heterozygous controls in the white light condition (test groups no. 1–10). However, we observed no courtship responses in the dim red light condition (test groups no. 11–20). Each column represents the mean of 18 tests. (**b**) The transmission of chemical signals was not necessary for PPL2ab neuron-mediated male-male courtship behaviour. The illustration of the device at the top of the panel for test groups no. 1–18 depicts a courtship testing arena with a transparent plastic paper, which blocked any possible transmission of chemical cues between courter and courtee males. There were significant differences in the courtship index between flies carrying *murashka-1-Gal4* > *UAS-TH* or *LG121-LexA* > *LexAop-TH* and their corresponding driver- and effector-heterozygous controls (test groups no. 1–6). When we used a dead (test groups no. 7–12) or immobilized decapitated male (test groups no. 13–18) as a courtee in the courtship pair test, we did not observe any courtship responses (# denotes no score for courtship index). When the dead courtee was moved manually in a motion without a fixed trajectory (the assay is diagrammed at the top of the test results for groups no. 19–24), the inter-male courtship behaviour was once more observed. We found significant differences in flies carrying *murashka-1-Gal4* > *UAS-TH* or *LG121-LexA* > *LexAop-TH* compared to their corresponding driver- and effector-heterozygous controls (test groups no. 19–24). Each column represents the mean of 18 tests. Error bars indicate + SEM, ^***^P *<* 0.001. We used a one-way ANOVA followed by a Tukey’s test for our analyses.

Courtship in *Drosophila* involves the exchange of various sensory stimuli, which are external cues processed by higher order brain centres to elicit suitable courtship responses. To analyse the relationship between external cues and PPL2ab neuron-mediated male-male courtship behaviour, we first blocked visual input by housing flies in a dim red light environment. When DA levels were increased in PPL2ab neurons in this dim red light environment, the inter-male courtship behaviour formerly observed in a white light environment (Figs 1e and 3a, test group nos 1–10) completely vanished (Fig. 3a; test group nos 11–20). The CI values measured under the two conditions were significantly different (Fig. 3a; test group nos 3 vs. 13, no. 5 vs. 15, no. 7 vs. 17, and no. 10 vs. 20). When the same test groups were returned to the white light condition, inter-male courtship was observed once again (data not shown). The above results confirm that visual cues are necessary to initiate PPL2ab neuron-mediated inter-male courtship behaviour.

We then investigated whether chemical cues affect male-male courtship behaviour. We set a transparent plastic paper down in the pair-testing arena to block any possible transmission of chemical signals between courter and courtee males (devices at top of Fig. 3b). Under white light conditions, males with high DA levels in PPL2ab neurons still exhibited courtship behaviour toward CS males (Fig. 3b, test groups nos 1–6, Supplementary Movie S3), confirming that chemical cues are not essential for initiating PPL2ab neuron-mediated inter-male courtship behaviour. Interestingly, when chemical cues were blocked and the target male was dead (Fig. 3b, test groups nos 7–12) or immobilized via decapitation (Fig. 3b, test groups nos 13–18), no inter-male courtship behaviour was observed following any of our genetic manipulations. These findings further suggest that PPL2ab neuron-mediated inter-male courtship behaviour is initiated by motion cues from target males. Indeed, even the input of motion cues through the manual movement of a dead courtee in a pattern without a fixed trajectory restored courtship responses (Fig. 3b, test groups nos 19–24, Supplementary Movie S4). Taken together, these results suggest that visually perceived motion signals may be important for initiating PPL2ab neuron-mediated male-male courtship behaviour.

### Reductions in DA levels in PPL2ab neurons enhance the attractiveness of males to other males

**Figure 4:**
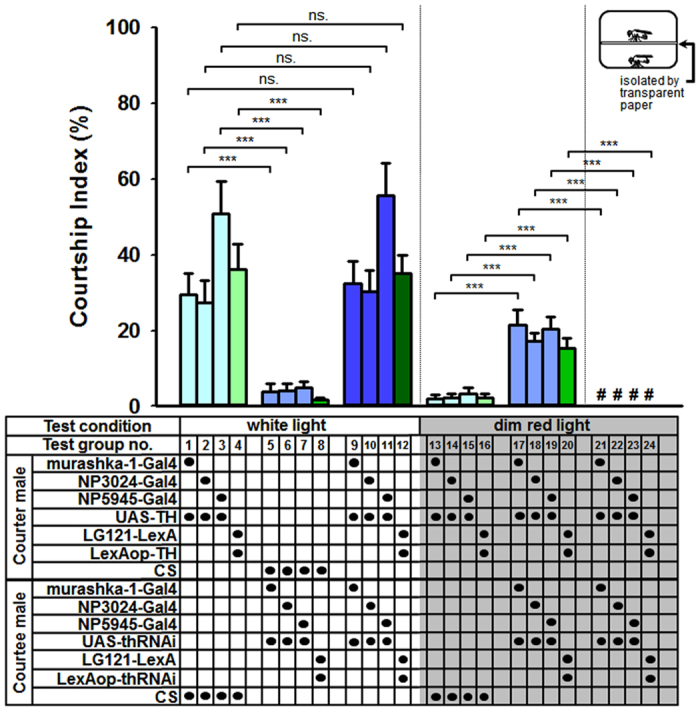
Reduction of DA levels in PPL2ab neurons enhances the attractiveness of males to other males. Courter males with high DA in PPL2ab neurons (*murashka-1-Gal4* > *UAS-TH, NP3024-Gal4* > *UAS-TH, NP5945-Gal4* > *UAS-TH*, or *LG121-LexA* > *LexAop-TH*) demonstrated courtship responses toward CS courtee males (test groups nos 1–4). However, there was no courtship response in CS courter males toward courtee males with low DA levels in their PPL2ab neurons (*murashka-1-Gal4* > *UAS-thRNAi, NP3024-Gal4* > *UAS-thRNAi, NP5945-Gal4* > *UAS-thRNAi*, and *LG121-LexA* > *LexAop-thRNAi*; test groups no. 5–8). When males with high DA levels were used as courters and males with low DA levels in PPL2ab neurons were used as courtees (test groups no. 9–12), there was a non-significant enhancement in the courtship responses (test group no. 9 vs. 1, test group no. 10 vs. 2, test group no. 11 vs. 3, and test group no. 12 vs. 4; ns: *P* > 0.05). Conducting the same experiments in a dim red light environment (test groups no. 13–24) revealed a significant difference in the courtship index (test group no. 13 vs. 17, test group no. 14 vs. 18, test group no. 15 vs. 19, and test group no. 16 vs. 20; *P* *<* 0.001). When we performed a pair test in the courtship testing arena with a transparent plastic paper to block any possible transmission of chemical signals between the courter and courtee (the device is illustrated at the top of the diagram for test groups no. 21–24), no male-male courtship behaviour was observed in any of the flies (# denotes no score for the courtship index). We observed significant differences in the courtship index compared to the same flies tested in an ordinary courtship arena (test group no. 21 vs. 17, test group no. 22 vs. 18, test group no. 23 vs. 19, and test group no. 24 vs. 20; *P* *<* 0.001). Each column represents the mean of 18 tests. Error bars indicate + SEM, ^***^P *<* 0.001. Analyses were by a one-way ANOVA followed by Tukey’s test.

It is known that when DA is decreased in male *Drosophila*, it can prompt inter-male courtship behaviour in wild-type males^5^. Here, we observed inter-male courtship behaviour when DA levels were specifically elevated in PPL2ab neurons (Figs 1e and 4, test groups nos 1–4). To determine whether the down-regulation of DA in PPL2ab neurons leads to attraction to CS males and stimulates male-male courtship behaviour, we used RNA interference to inhibit TH expression and thereby reduce DA levels in PPL2ab neurons (*murashka-1-Gal4* > *UAS-thRNAi, NP5945* > *UAS-thRNAi, NP3024* > *UAS-thRNAi*, and *LG121* > *LexAop-thRNAi*). Using this approach, we observed that CS courter males do not initiate inter-male courtship behaviour in target males (Fig. 4, test groups nos 5–8). There was, however, a significant alteration in CI values of courter males with increased DA levels in PPL2ab neurons toward CS male courtees (Fig. 4; test group no. 1 vs. 5, no. 2 vs. 6, no. 3 vs. 7, and no. 4 vs. 8). We found no significant differences in the CIs of courter males with increased DA levels in PPL2ab neurons toward courtee males with decreased DA levels in PPL2ab neurons or wild-type CS males (Fig. 4; test group no. 1 vs. 9, no. 2 vs. 10, no. 3 vs. 11, and no. 4 vs. 12).

Based on these results, we initially speculated that males with decreased DA in PPL2ab neurons are unable to persuade CS males to initiate male-male courtship behaviour. As previously mentioned, when male flies are placed in an environment lit by a dim red light, increased DA levels in PPL2ab neurons in males has no discernible effect on courtship toward CS males (Figs 3a and 4, test groups nos 13–16). Unexpectedly, we observed courtship behaviour in males with high DA levels in PPL2ab neurons towards males with low DA levels in PPL2ab neurons (Supplementary Movie S5). This observation is reflected in a significant difference between the CIs of the two groups (Fig. 4; test groups no. 13 vs. 17, no. 14 vs. 18, no. 15 vs. 19, and no. 16 vs. 20). Therefore, we can conclude that males with decreased DA in PPL2ab neurons faithfully attract males with high DA in their PPL2ab neurons. This in turn results the inter-male courtship behaviour. Our interpretation of this finding is that courtship intensity toward CS males reaches its maximal level after DA is increased in PPL2ab neurons. Therefore, by blocking visual cues during courtship and eliminating the ceiling effect, we were able to demonstrate that compared to CS courtees, there is a significant increase in CI in the presence of a male courtee with low DA levels in PPL2ab neurons. Further studies in the dim red light condition and the use of transparent plastic paper to block the transmission of chemical cues between courter and courtee males eliminated inter-male courtship behaviour between courter males with high DA levels in their PPL2ab neurons and courtee males with low DA levels in their PPL2ab neurons (Fig. 4, test groups nos 21–24).

### Low DA levels in PPL2ab neurons act through CH pheromones associated with male attractiveness

**Figure 5:**
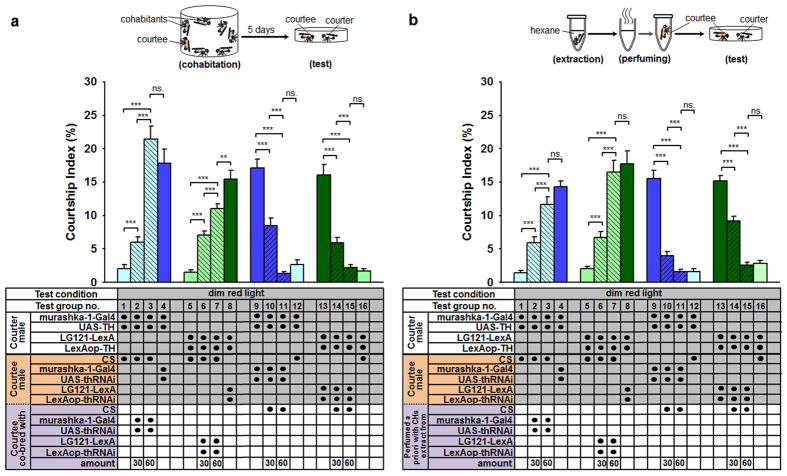
Reduced PPL2ab neuron DA enhances the attractiveness of males to other males via changes in CHs. (**a**) After 10 CS courtee males were bred together with 30 or 60 males with low DA levels in their PPL2ab neurons for 5 days, we observed a significant attraction of courter males with high DA levels in their PPL2ab neurons to the CS males (test groups no. 2–3 and 6–7) (test groups no. 2 or 3 vs. 1, test groups no. 6 or 7 vs. 5; *P* *<* 0.001). Contrarily, when the 10 courtee males with low DA levels in their PPL2ab neurons were bred together with 30 or 60 CS males for 5 days, the treated males were observed to be less attractive to courter males with high PPL2ab neuron DA levels (test groups no. 10–11 and 14–15) (test groups no. 10 or 11 vs. 9, test groups no. 14 or 15 vs. 13; *P* *<* 0.001). (**b**) When 10 CS courtee males with low DA levels in their PPL2ab neurons were perfumed with CH extracts from 30 or 60 other males, we observed a significant increase in the attractiveness of the treated CS males to courter males with high DA levels in their PPL2ab neurons (test groups no. 2–3 and 6–7) (test groups no. 2 or 3 vs. 1, test groups no. 6 or 7 vs. 5; *P* *<* 0.001). Contrarily, when 10 courtee males with low PPL2ab DA levels were perfumed with CH extracts from 30 or 60 CS males, we observed that the treated males were less attractive to courter males with high PPL2ab neuron DA (test groups no. 10–11 and 14–15; test groups no. 10 or 11 vs. 9, test groups no. 14 or 15 vs. 13; *P* *<* 0.001). Each column represents the mean of 18 tests. Error bars indicate + SEM, ^***^P *<* 0.001, ^**^P *<* 0.01. Analyses were by a one-way ANOVA followed by Tukey’s test.

We used two different strategies to prove that males with low DA levels attract males with high DA levels in PPL2ab neurons via the expression of CHs. First, 10 CS males that were set aside as courtees were bred with 30 or 60 males with low levels of DA in their PPL2ab neurons in a vial (2.5 cm diameter × 2.5 cm high) for 5 days post-eclosion. We observed male-male courtship under a dim red light. We found that males with high DA levels are attracted to CS males that had been co-bred with males with low DA levels in their PPL2ab neurons (Fig. 5a; test groups nos 2, 3, 6, and 7). The CIs (Fig. 5a; test groups no. 1 vs. 2 or 3, and test groups no. 5 vs. 6 or 7) in the different test groups were significantly different when compared to those of untreated CS male targets. Interestingly, the CIs of males that had cohabitated with males with decreased DA increased significantly and proportionally to the number of CS male cohabitants (i.e., 0, 30, or 60) (Fig. 5a, test groups nos 1–3 and 5–7). We also took 10 future courtee males with low DA in their PPL2ab neurons and bred them with 30 or 60 CS males in a vial (2.5 cm diameter × 2.5 cm high) for 5 days post-eclosion. In the dim red light condition, courtship responses in males with high DA levels toward males with low DA levels in PPL2ab neurons that had been co-bred with CS males were down-regulated (Fig. 5a; test groups no. 10, 11, 14, and 15). Moreover, the CIs of these flies (Fig. 5a; test groups no. 9 vs. 10 or 11, and 13 vs. 14 or 15) were significantly different from those of courtee males with low DA levels in their PPL2ab neurons. Interestingly, both of these groups had CIs that were significantly decreased in a directly proportional manner when we evaluated males that had been bred with 0, 30, or 60 CS male cohabitants (Fig. 5a; test groups nos 9–11 and nos 13–15).

To avoid the effects of social experience during co-breeding on the following courtship analysis, we washed out CHs from the 30 and 60 males with low DA levels in PPL2ab neurons by exposing them to 500 μl of hexane and applied these extracted CHs to 10 other CS males. Under dim red light, we observed that males with high DA levels in PPL2ab neurons were apparently attracted to the CH-treated CS males (Fig. 5b, test groups nos 1–8). The CIs of these flies (Fig. 5c, test groups nos 2–3 and nos 6–7) significantly increased as a function of the number of males whose CHs were washed out. In contrast, if CHs from the 30 and 60 CS male vials were washed out with 500 μl of hexane and subsequently applied to males with low DA levels in PPL2ab neurons, we observed that males that had been perfumed with the extracted CHs from CS males were significantly less attractive to males with high DA in PPL2ab neurons (Fig. 5b, test groups nos 9–16). The CIs of these lies also significantly decreased with increasing numbers of CS males whose CHs had been washed out (Fig. 5b, test groups nos 10–11 and 14–15). Thus, we speculate that the extracted CHs have unusual compositions due to feminization in males with low DA levels in their PPL2ab neurons. Moreover, we suspect that the change in composition may have included an increase in female sex pheromones (Fig. 5b, test groups nos 2–3 and 6–7) and a decrease in male sex pheromones, which typically repel other males as mates (Fig. 5b, test groups nos 10–11 and nos 14–15). Indeed, these hypotheses were verified when we treated CS males with CHs from males with decreased DA levels in their PPL2ab neurons or when we applied the CHs of CS males onto males with decreased DA in order to alter their attractiveness to males with high DA in PPL2ab neurons.

## Discussion

In characterizing the four drivers (*murashka-1-, NP3024-, NP5945-Gal4*, and *LG121-LexA*), we observed that only TH-positive PPL2ab neurons were labelled in all four independent drivers (Fig. 1a–d). Indeed, the expression patterns of the four drivers are very broad. This makes it difficult to conclude that PPL2ab neurons are the neurons responsible for the observed effects. Therefore, we used intersectional genetic strategies to precisely overexpress TH in PPL2ab neurons, which led to an enhanced male-to-male courtship index (Fig. 1i). These results indicate that increased DA is sufficient to induce male-male courtship behaviour within this particular subset of PPL2ab neurons. Nevertheless, to reduce the possibility of negligence, we also compared the expression patterns of the 4 drivers (*murashka-1-, NP3024-, NP5945-*Gal4 and *LG121-LexA*) in the adult ventral nerve cord (VNC) and peripheral chemosensory tissues, including the proboscis, antenna, and foreleg. These tissues have chemical receptors used in the modulation of courtship responses by chemosensory perception. The findings are clearly presented that three Gal4 drivers (*murashka-1-, NP3024-*, and *NP5945-Gal4*) are mainly expressed in the proboscis and the VNC, while the *LG121-LexA* driver was not expressed in the above-mentioned regions (Supplementary Fig. S1a–d). We also could not detect any colocalization of *murashka-1-Gal4* and TH expression in neuronal cell bodies in the VNC (Supplementary Figs S1h1–3). For the assertion of the specific manipulation in the PPL2ab neurons using these drivers in our study, no additional GAL4-expression should be colocalized in these regions.

Previous studies have shown that increasing DA levels in DA-expressing cells (*TH-Gal4* > *UAS-TH*) can induce *Drosophila* male-to-male courtship behaviour^4^. We found that changes in DA levels in a specific population of DAergic PPL2ab neurons in the male *Drosophila* brain prompts inter-male courtship behaviour. Interestingly, we found no significant differences in CIs and ChIs between males with increased DA levels in PPL2ab neurons (*murashka-1-Gal4* > *UAS-TH, NP5945-Gal4* *>* *UAS-TH, NP3024-Gal4* > *UAS-TH*, and *LG121-LexA* > *LexAop-TH*) and those with increased DA in almost all DA cells (*TH-Gal4* > *UAS-TH*). These findings imply that inter-male courtship behaviour caused by high DA levels is mainly modulated by PPL2ab neurons (Fig. 1e and 1f).

When the communication of chemical signals between the courter and courtee was blocked, male-male courtship behaviour was still observed in males with high DA levels in PPL2ab neurons (Fig. 3b, test groups nos 1–6). In contrast, inter-male courtship behaviour was not observed in an environment with dim red light (Fig. 3a). This suggests that visual cues play a more important role than chemical cues in PPL2ab neuron-prompted male-male courtship behaviour. We further confirmed that motion cues contribute to the initiation of PPL2ab neuron-prompted inter-male courtship behaviour (Fig. 3b). It has been shown that blocking acoustic, visual, olfactory, and taste inputs can inhibit the inter-male courtship behaviour induced by the overexpression of TH in DAergic neurons (*TH-Gal4* > *UAS-TH*)^4^. However, we found that DA-induced inter-male courtship behaviour brought on through the specific manipulation of PPL2ab neurons was only affected by the blockage of visual signal input. It has been suggested that fruitless (fru)- and doublesex (dsx)-positive P1 neurons receive chemosensory cues and enhance courtship responses to moving objects^17^,^18^. The mechanisms underlying male-male courtship behaviour induced by visual cues via PPL2ab neural circuitry, and whether these cues are associated with P1 neural circuitry requires further study.

Previous studies have also demonstrated male-male courtship behaviour can be initiated via decreases in male DA levels^5^. In our study, although inter-male courtship in *Drosophila* was observed in males with high DA levels in PPL2ab neurons, decreased DA levels in PPL2ab neurons did not elicit other wild-type CS males to perform male-male courtship behaviour (Fig. 4, test groups nos 5–8). There were also no significant differences between CIs in males with high DA levels in PPL2ab neurons toward males with low DA levels in PPL2ab neurons and those toward wild-type CS males (Fig. 4a, test groups nos 1–4 vs. 9–12). Interestingly, when visual cues were blocked, the existing inter-male courtship behaviour in males with high DA levels in PPL2ab neurons toward CS males was inhibited (Fig. 4a, test groups nos 13–16). However, if courtees were targeted by males with low DA levels in PPL2ab neurons; male-to-male courtship behaviour was still observed (Fig. 4, test groups nos 17–20). This observation indicates that males with low DA levels attract courter males with high DA levels in PPL2ab neurons. Most compellingly, when DA levels were decreased in PPL2ab neurons, inter-male courtship behaviour appeared to occur via changes in cuticular pheromones (Fig. 5b). We observed that CS males perfumed with extracted CHs from males with low DA levels in PPL2ab neurons had apparent sex appeal to the courter flies (Fig. 5b, test groups nos 1–8). In contrast, males with low DA levels in PPL2ab neurons that were perfumed with the extracted CHs from CS males apparently lost their sex appeal for the courter flies (Fig. 5b, test groups nos 9–16). These observations indicate that the CHs from males with low DA in PPL2ab neurons may have reduced inhibitory chemical signals and increased encouraging chemical signals, leading to higher sex appeal on. According to our GC-MS results, we confirmed that none of our three genotypes of males (carrying *murashka-1-Gal4* > *UAS-thRNAi* and their corresponding heterozygous controls) produced detectable amounts of the two major female specific cuticular compounds, 7,11-heptacosadiene (7,11-HD) and 7,11-nonacosadiene (7,11-ND). All three types of males have the same major CHs, including cis-Vaccenyl acetate (cVa), alkane and alkene, which are well-known in many published studies. These three types of males also reveal very similar gas chromatograms (Supplementary Fig. S2). Table shows the relative amounts (where mean proportions were calculated from peak areas) of 18 cuticular compounds (Supplementary Table S1). There were no significant differences among male types on cVa (#2), 7-tricosene (7-T; #6), and 7-pentacosene (7-P; #13), the three important compounds known for their male-male communication roles. Thus, changes in these 5 CHs do not appear to underlie our observations. Significant differences were only found on three (#1, #4 and #9) of the 18 chemicals, and their relative amounts were all lower than only 3%. Therefore, we concluded that neither the quality nor the relative quantity of the CHs with larger amounts were significantly different among the males with low DA in PPL2ab neurons and relative controls. However, it is possible that our observations are due to changes in previously undescribed minor sex pheromones. We suspected that the major differences among these different types of males may lie in some other chemicals waiting to be identified. More specifically, male-male attraction only occurred when a male with increased DA in PPL2ab neurons was the courter. CS male courters were not attracted (Fig. 4, test groups nos 13–16) to other males. We therefore propose that males with high sex drive (those with a lower courtship response threshold), such as those with increased DA in their PPL2ab neurons, may have higher sexual motivation. Moreover, these males may sense small changes in CHs in males with decreased DA levels in their PPL2ab neurons. Recent reports indicate that male courtship vigour is correlated with Or47b olfactory neuron sensitivity to pheromones and that juvenile hormones directly enhance the pheromone sensitivity of these neurons^19^. However, whether PPL2ab neurons simply alter the sensitivity/specificity of a pheromone sensing system is still unknown.

In analysing PPL2ab neuron-prompted inter-male courtship behaviour, we found that the courtship responses of males with high PPL2ab neuron DA toward wild-type CS females were significantly higher than toward CS males (Fig. 2). Nevertheless, PPL2ab neuron-prompted male-male courtship is triggered primarily by the target’s motion signals. We wondered whether we misjudged these males in thinking that they do not alter their preference for females, as they lose interest in “decapitated (immobilized) male competitors”. However, having an “intact male” and an “intact female” is a must. We therefore only used “intact” flies as targets for the competition test. These results also prove males do not change their preference for females when they have increased DA levels in their PPL2ab neurons (Supplementary Fig. S3a). However, it is paradoxical that there is a minor male-male CI score when a tester male is exposed to an immobilized target male in the competition test (Fig. 2). Is male-male courtship prompted by DA in PPL2ab neurons definitely encouraged by the target’s motion? Both competitors of intact CS males and decapitated CS males are used as targets for the competition test. We found that “tester males” obviously exhibit stronger courtship toward intact (movable) males (Supplementary Fig. S3b). In sum, we demonstrate that males with up-regulated DA do not change their preference toward females. This may in turn lead to motion-cue-dependent male-male courtship.

## Methods

### Fly strains

Fly stocks were raised on standard cornmeal food and housed at 25 °C and 70% relative humidity on a 12:12 hour light:dark (L/D) cycle. The wild-type strain used was Canton-S w (CS10). The GAL4 and LexA driver strains used were *TH-Gal4*^20^, *murashka-1-Gal4, NP3024-Gal4, NP5945-Gal4*^2^ and *LG121-LexA* (a chimeric LexA::GAL4AD driver)^2^. The *UAS*- and *LexAop*-effector strains used were *UAS–TH*^21^, *UAS-thRNAi* (#108879; purchased from the Vienna *Drosophila* Resource Center, Vienna, Austria), *UAS*–*mCD8::GFP* (#5137; purchased from the Bloomington *Drosophila* Stock Center, University of Indiana, IN, USA), *UAS-LexPR* and *LexAop-GeneSwitch*^16^, *LexAop-TH, LexAop-thRNAi, LexAop-FLP*, and *UAS-frt-stop-frt-TH*^2^. All behavioural analyses using transgenic expression utilized the progeny obtained from crossing Gal4 or LexA flies crossed to those with the indicated reporter or effector transgenes.

### Immunohistochemistry and imaging

Whole-mount immunolabeling of the adult brain was performed as previously described^2^.

### Behavioural assays

Naive males with no pretested social experience were collected on the day of eclosion and kept in individual test tubes in a 25 °C incubator with a 12-hour L/D cycle. Target males were stored in groups (e.g., 20 males per vial). Courtship assays were performed between hours 2 and 6 of the light cycle every day. Paired courtship chambers (1.2 cm diameter × 0.8 cm high) containing a layer of yeast food were assembled in each cell. Both test males and target males were transferred (3 days post-eclosion) to each cell of the chamber for pair-testing and video recording (HDR-SR10 digital video camera, Sony, Japan) for 10 minutes. The CI was determined by calculating the percentage of the 10-minute recording period wherein the courter male courted the courtee male (i.e., tapping, following, wing vibration, and attempted copulation)^2^. Male-male courtship usually involves the close following, or chaining, of males. In these cases, the flies form something resembling a conga-like line. As the chain slinks around, the flies can exhibit other aspects of courtship, such as wing extensions. The mutant allele *fru*^1^ originally led to the observation of chain formation^22^. We used 10 naive males in a new chamber (9.5 cm diameter × 0.8 cm high) containing a layer of yeast food to perform each chaining assay. A chain was defined as a group of at least three males exhibiting courtship behaviour toward each other^23^. The ChI was determined by calculating the percentage of time that groups of males spent courting during a 10-minute observation period. A competitive courtship assay was performed with a single tester male simultaneously presented with two different competitor target flies in a courtship chamber (1.2 cm diameter × 0.8 cm high). CIs toward each target were simultaneously measured and then compared^24^. All courtship assays were scored by two observers blind to genotype and treatment.

### Fly co-breeding and perfuming

#### Fly cohabitation

Ten CS males were bred together with 30 or 60 males with low DA levels in their PPL2ab neurons (*murashka-1-Gal4* > *UAS-thRNAi*, and *LG121-LexA* > *LexAop-thRNAi*) in a glass vial (2.5 cm diameter × 2.5 cm high) for 5 days. Alternatively, 10 males with low DA levels in their PPL2ab neurons (*murashka-1-Gal4* > *UAS-thRNAi*, and *LG121-LexA* > *LexAop-thRNAi*) were bred together with 30 or 60 CS males, which enabled the transfer of chemical cues among all cohabitants. Subsequently, the 10 treated flies were used as courtee males and those with high DA levels in PPL2ab neurons (*murashka-1-Gal4* > *UAS-TH*, and *LG121-LexA* > *LexAop-TH*) were used as courter males in courtship pair tests.

#### Fly perfuming

Thirty or 60 male flies (CS, *murashka-1-Gal4* > *UAS-thRNAi*, and *LG121-LexA* > *LexAop-thRNAi*) were soaked in 0.5 ml of hexane (Sigma Aldrich) for 10 minutes. The resulting solution was then pipetted into a glass vial (2.5 cm diameter × 2.5 cm high). The hexane was then evaporated under nitrogen gas, leaving the CH compounds as a residue coating the bottom of the vial. Groups of 10 CS male flies were transferred to vials containing the CH-coated (*murashka-1-Gal4* > *UAS-thRNAi*, or *LG121-LexA* > *LexAop-thRNAi*) vials. Alternatively, 10 males with low DA levels in their PPL2ab neurons (*murashka-1-Gal4* > *UAS-thRNAi*, and *LG121-LexA* > *LexAop-thRNAi*) were transferred to the vial containing CS CH-coated males. Subsequently, all vials were subjected to three medium vortex pulses lasting 20 seconds, with 20-second pauses between each pulse. This process did not seem to harm the flies. The treated flies were then transferred to a fresh vial with food medium and allowed to recover and groom for 1 hour^25^. They were then used as courtee males in further courtship pair tests with courter males with high DA levels in PPL2ab neurons (*murashka-1-Gal4* > *UAS-TH*, and *LG121-LexA* > *LexAop-TH*).

### Statistical Analysis

Statistical analyses were performed using SigmaPlot software version 12.0. All data were evaluated via one-way analyses of variance (ANOVAs) followed by Tukey’s tests or two-way ANOVAs followed by Bonferroni multiple-comparisons tests. All data are presented as means + standard errors of the mean (SEMs).

## Additional Information

**How to cite this article:** Chen, S.-L. *et al*. Active and passive sexual roles that arise in *Drosophila* male-male courtship are modulated by dopamine levels in PPL2ab neurons. *Sci. Rep.* **7**, 44595; doi: 10.1038/srep44595 (2017).

**Publisher's note:** Springer Nature remains neutral with regard to jurisdictional claims in published maps and institutional affiliations.

## Supplementary Material

Supplementary Movie S1

Supplementary Movie S2

Supplementary Movie S3

Supplementary Movie S4

Supplementary Movie S5

Supplementary Information
