# Supplementary material for: Active and passive sexual roles that arise in Drosophila male-male courtship are modulated by dopamine levels in PPL2ab neurons
Source: Sci Rep. 2017 Mar 15;7:44595. doi: 10.1038/srep44595 (PMC5353583; doi:10.1038/srep44595)
Supplement: Supplementary Information [file srep44595-s6.doc]

**Supplemental Information**

**Active and passive sexual roles that arise in Drosophila male-male courtship are modulated by dopamine levels in PPL2ab neurons**

Shiu-Ling Chen, Yu-Hui Chen, Chuan-Chan Wang, Yhu-Wei Yu, Yu-Chen Tsai, Hsiao-Wen Hsu, Chia-Lin Wu, Pei-Yu Wang, Lien-Cheng Chen, Tsuo-Hung Lan, Tsai-Feng Fu

**Supplementary Inventory**

1. **Supplementary Videos**
2. **Supplementary Table**
3. **Supplementary Figures**
4. **Genotypes**
5. **Supplementary Videos**

**Movie S1.** *murashka-1-Gal4/UAS-TH* male courting a wild-type CS male

**Movie S2.** *murashka-1-Gal4/UAS-TH* [males forming courtship chains](http://www.sciencedirect.com/science/MiamiMultiMediaURL/1-s2.0-S0092867405004071/1-s2.0-S0092867405004071-mmc3.mov/272196/html/S0092867405004071/db426ca20605f303ea2f96f9a9ee5ee6/mmc3.mov)

**Movie S3.** *murashka-1-Gal4/UAS-TH* male courting a wild-type CS male when chemical signals are blocked

**Movie S4.** *murashka-1-Gal4/UAS-TH* male courting a wild-type manually motive dead CS male when chemical signals are blocked

**Movie S5.** *murashka-1-Gal4/UAS-TH* male courting a *murashka-1-Gal4/UAS-thRNAi* male in a dim red light environment

1. **Supplementary Table**

| **Table S1.** Mean proportions (±SD) of the CHs extracted from M1 (*UAS-thRNAi/+;murashka-1-Gal4/+*), M2 (*+/+;murashka-1-Gal4/+*), and M3 (*UAS-thRNAi/+;+/+*) of *D. melanogaster* male. Proportions were calculated basing on the total peak areas of the 18 chemicals listed in the table. The effects of male type on proportions of each compound were analyzed by ANOVA and Tukey's test. Same letters indicate significant differences between two male types on a specific chemical. | | | | | | | | | |
| --- | --- | --- | --- | --- | --- | --- | --- | --- | --- |
| # | Abbrev. | Compound Name | M1 | (n=6) | M2 | (n=6) | M3 | (n=5) | *p*-value (ANOVA) |
| 1 | *n*-C21 | *n*-Heneicos**a**ne | 0.008 | (±0.0011) | 0.006 | (±0.0004) a | 0.009 | (±0.0019) a | **0.029** |
| 2 | cVa | (Z)-11-Vaccenyl acetate | 0.054 | (±0.0245) | 0.049 | (±0.0394) | 0.059 | (±0.0192) | 0.683 |
| 3 | 7-D | (Z)-7-Docosene | 0.005 | (±0.0003) | 0.005 | (±0.0005) | 0.004 | (±0.0003) | 0.109 |
| 4 | *n*-C22 | *n*-Docosane | 0.008 | (±0.0004) ab | 0.005 | (±0.0003) ac | 0.007 | (±0.0005) bc | **<0.001** |
| 5 | 9-T | (Z)-9-Tricosene | 0.027 | (±0.0031) | 0.027 | (±0.0026) | 0.026 | (±0.0011) | 0.735 |
| 6 | 7-T | (Z)-7-Tricosene | 0.384 | (±0.0303) | 0.381 | (±0.0273) | 0.346 | (±0.0198) | 0.101 |
| 7 | 5-T | (Z)-5-Tricosene | 0.048 | (±0.0025) | 0.051 | (±0.0035) | 0.048 | (±0.0037) | 0.329 |
| 8 | *n*-C23 | *n*-Tricosane | 0.104 | (±0.0049) | 0.107 | (±0.0099) | 0.115 | (±0.0095) | 0.163 |
| 9 | 8-Te | (+)-8-Tetracosene | 0.007 | (±0.0007) ab | 0.004 | (±0.0008) a | 0.005 | (±0.0006) b | **<0.001** |
| 10 | 7-Te | (Z)-7-Tetracosene | 0.007 | (±0.0004) | 0.007 | (±0.0005) | 0.007 | (±0.0004) | 0.163 |
| 11 | 25-Br | 2-Methyltetracosane | 0.041 | (±0.0036) | 0.044 | (±0.0042) | 0.046 | (±0.0020) | 0.159 |
| 12 | 9-P | (Z)-9-Pentacosene | 0.022 | (±0.0012) a | 0.027 | (±0.0035) ab | 0.022 | (±0.0026) b | **0.010** |
| 13 | 7-P | (Z)-7-Pentacosene | 0.142 | (±0.0065) | 0.144 | (±0.0182) | 0.153 | (±0.0192) | 0.577 |
| 14 | 5-P | (Z)-5-Pentacosene | 0.005 | (±0.0007) | 0.005 | (±0.0012) | 0.007 | (±0.0022) | 0.093 |
| 15 | *n*-C25 | *n*-Pentacosane | 0.016 | (±0.0012) | 0.019 | (±0.0054) | 0.018 | (±0.0016) | 0.311 |
| 16 | 27-Br | 2-Methylhexacosane | 0.070 | (±0.0077) | 0.070 | (±0.0081) | 0.082 | (±0.0073) | 0.061 |
| 17 | *n*-C27 | *n*-Heptacosane | 0.005 | (±0.0008) | 0.008 | (±0.0051) | 0.006 | (±0.0006) | 0.431 |
| 18 | 29-Br | 2-Methyloctacosane | 0.047 | (±0.0062) | 0.041 | (±0.0127) | 0.041 | (±0.0036) | 0.378 |

1. **Supplementary Figures**

**Fig. S1 Characterization of the LexA and Gal4 drivers used in this study.** Tissues from 10-day-old adult males, including the antenna, proboscis, foreleg, and the ventral nerve cord (VNC) expressing the (a-d) *LG121-LexA*, (e-h) *murashka-1-Gal4*, (i-l) *NP5945-Gal4*, and (m-p) *NP3024-Gal4* drivers*,* reported by *LexAop-mCD8::GFP* or *UAS-mCD8::GFP* (green in a1-p1) were immunostained using an anti-TH antibody to label the DA cells of the VNC (magenta in d2, h2, i2, and p2). The expression pattern of each driver was assessed. The spaced arrowheads denote *NP5945-Gal4* and *NP3024-LexA* driver expression patterns reported by *UAS-mCD8::GFP* in the VNC colocalized with TH-positive neuronal cell bodies (in l3 and p3). The scale bar is 20 μm.

**Fig. S2 Reassessment of cuticular compounds in *D. melanogaster* males by** **Gas chromatography mass spectrometry (GC/MS).** We used a Shimadsu GC-2014 Gas Chromatograph with a DB-1HT for gas chromatograms of individual male flies carrying (a) *murashka-1/UAS-thRNAi* and their corresponding (b) driver- and (c) effector-heterozygous controls to calculate relative amounts of CHs and a Thermo DSQII GC/MS with a DB-1HT column to confirm the identity of each cuticular compound from gas chromatograms. Virgin 5-day-old flies were extracted by *n*-hexane individually for GC samples, and pooled for more than 10 individuals for the GC-MS samples. The MS-characterized compounds are listed in the Supplementary Table S1.

**Fig. S3 Precise testing of sexual preferences of males with increased DA in PPL2ab neurons to various conditional competitors.** Sexual preference determined by courtship competition tests indicated that (a) courter males carrying the *murashka-1-Gal4>UAS-TH* and *LG121-LexA>LexAop-TH* constructs had significantly higher male-to-intact-female CIs than male-to-intact-male CIs. (b) The courter males carrying the *murashka-1-Gal4>UAS-TH* construct significantly preferred intact target males compared to decapitated (immobilized) males. Each column represents the mean of 18 competitive courtship assays. Error bars indicate + SEM, *^***^P* < 0.001. Statistical analyses were by one-way ANOVAs followed by Tukey’s tests.

1. **Genotypes**

**Fig. 1**

1.) *+/y;+/UAS-mCD8::GFP;murashka-1-Gal4/ UAS-mCD8::GFP*

2.) *+/y;+/UAS-mCD8::GFP;NP3024-Gal4/UAS-mCD8::GFP*

3.) *+/y;+/UAS-mCD8::GFP;NP5945-Gal4/UAS-mCD8::GFP*

4.) *+/y;+/LexAop-mCD8::GFP;LG121-LexA/+*

5.) *+/y;+/+;TH-Gal4/UAS-TH*

6.) *+/y;+/+;TH-Gal4/+*

7.) *+/y;+/+;murashka-1-Gal4/UAS-TH*

8.) *+/y;+/+;murashka-1-Gal4/+*

9.) *+/y;+/+;NP3024-Gal4/UAS-TH*

10.) *+/y;+/+;NP3024-Gal4/+*

11.) *+/y;+/+;NP5945-Gal4/UAS-TH*

12.) *+/y;+/+;NP5945-Gal4/+*

13.) *+/y;+/+;+/UAS-TH*

14.) *+/y;LexAop-TH/+;LG121/+*

15) *+/y;+/+;LG121/+*

16) *+/y;LexAop-TH/+;+/+*

17.) *+/y;UAS-LexPR/+;murashka-1-Gal4/LexAop-TH*

18.) *+/y;UAS-LexPR/+;murashka-1-Gal4/+*

19.) *LexAop-FLP/y; UAS>*>TH/+; LG121/murashka-1-Gal4*

20.) *LexAop-FLP/y;+; murashka-1-Gal4/+*

21.) +*/y; UAS>*>TH/+; LG121+*.

**Fig. 2**

1.) *+/y;+/+;murashka-1-Gal4/UAS-TH*

2.) *+/y;+/+;murashka-1-Gal4/+*

3.) *+/y;+/+;NP3024-Gal4/UAS-TH*

4.) *+/y;+/+;NP3024-Gal4/+*

5.) *+/y;+/+;NP5945-Gal4/UAS-TH*

6.) *+/y;+/+;+/UAS-TH*

7.) *+/y;LexAop-TH/+;LG121/+*.

**Fig. 3**

1.) *+/y;+/+;murashka-1-Gal4/UAS-TH*

2.) *+/y;+/+;murashka-1-Gal4/+*

3.) *+/y;+/+;NP3024-Gal4/UAS-TH*

4.) *+/y;+/+;NP3024-Gal4/+*

5.) *+/y;+/+;NP5945-Gal4/UAS-TH*

6.) *+/y;+/+;NP5945-Gal4/+*

7.) *+/y;+/+;+/UAS-TH*

8.) *+/y;LexAop-TH/+;LG121/+*

9) *+/y;+/+;LG121/+*

10) *+/y;LexAop-TH/+;+/+.*

**Fig. 4**

1.) *+/y;+/+;murashka-1-Gal4/UAS-TH*

2.) *+/y;+/+;NP3024-Gal4/UAS-TH*

3.) *+/y;+/+;NP5945-Gal4/UAS-TH*

4.) *+/y;LexAop-TH/+;LG121/+*

5.) *+/y;+/+;murashka-1-Gal4/UAS-thRNAi*

6.) *+/y;+/+;NP3024-Gal4/UAS-thRNAi*

7.) *+/y;+/+;NP5945-Gal4/UAS-thRNAi*

8.) *+/y;+/+;LG121/LexAop-thRNAi.*

**Fig. 5**

1.) *+/y;+/+;murashka-1-Gal4/UAS-TH*

2.) *+/y;+/+;NP3024-Gal4/UAS-TH*

3.) *+/y;+/+;NP5945-Gal4/UAS-TH*

4.) *+/y;LexAop-TH/+;LG121/+*

5.) *+/y;+/+;murashka-1-Gal4/UAS-thRNAi*

6.) *+/y;+/+;NP3024-Gal4/UAS-thRNAi*

7.) *+/y;+/+;NP5945-Gal4/UAS-thRNAi*

8.) *+/y;+/+;LG121/LexAop-thRNAi.*

**Fig. S1**

1.) *+/y;LexAop-mCD8::GFP/+; LG121*/+

2.) *+/y;UAS-mCD8::GFP/+; murashka-1/UAS-mCD8::GFP*

3.) *+/y;UAS-mCD8::GFP/+; NP5945/UAS-mCD8::GFP*

4) *+/y;UAS-mCD8::GFP/+; NP3024/UAS-mCD8::GFP.*

**Fig. S2**

1.) *+/y;+/+;murashka-1-Gal4/UAS-thRNAi*

2.) *+/y;+/+;murashka-1-Gal4/+*

3.) *+/y;+/+;+/UAS-thRNAi*

**Fig. S3**

1.) *+/y;+/+;murashka-1-Gal4/UAS-TH*

2.) *+/y;+/+;LG121/LexAop-thRNAi.*
